# RETRACTION: Protocatechuic Acid Prevents oxLDL‐Induced Apoptosis by Activating JNK/Nrf2 Survival Signals in Macrophages

**DOI:** 10.1155/omcl/9898245

**Published:** 2026-04-07

**Authors:** Oxidative Medicine and Cellular Longevity

RETRACTION: R. Varì, B. Scazzocchio, C. Santangelo, C. Filesi, F. Galvano, M. D’Archivio, R. Masella, and C. Giovannini, “Protocatechuic Acid Prevents oxLDL‐Induced Apoptosis by Activating JNK/Nrf2 Survival Signals in Macrophages,” *Oxidative Medicine and Cellular Longevity* 2015, no. 1 (2015): 1‐11, https://doi.org/10.1155/2015/351827.

The above article, published online on 9 June 2015 in Wiley Online Library (wileyonlinelibrary.com), has been retracted by agreement between the journal Chief Editor, Jeannette Vasquez‐Vivar; and John Wiley & Sons Ltd.

The retraction has been agreed upon following an investigation into concerns raised by readers on PubPeer [1], which identified multiple instances of duplicated and spliced Western blots in Figures 3A–D, 4B, 5A,B, and 6A–C, some of which appear to have been flipped or re‐sized. Due to the significant duplications and splicing identified, the data and conclusions of this article are considered unreliable.

The authors disagree with the retraction.
